# Anticipation of Stress and Relaxation Dynamically Impacts Sleep

**DOI:** 10.3390/clockssleep7040068

**Published:** 2025-12-03

**Authors:** Sandrine Baselgia, Jonas Beck, Björn Rasch

**Affiliations:** 1 Department of Psychology, Université de Fribourg, Rue P.-A. de Faucigny 2, 1700 Fribourg, Switzerland; 2 Swiss Sleep House Bern, Department of Neurology, University Hospital (Inselspital), University of Bern, 3010 Bern, Switzerland

**Keywords:** sleep, stress, cognition, arousal, slow waves

## Abstract

Anticipation of stressful events can impair sleep quality. In a recent study, we reported that anticipating a stressful task before a nap led to negative changes in sleep parameters, particularly at the end of the nap. In our previous study, we compared stress anticipation with the anticipation of relaxation; thus, the observed effects may have been amplified by sleep quality improvements in the relaxation condition. In the current study, we aimed to replicate these findings using an alternative neutral control condition. The data from a newly collected sample (*n* = 31) were compared with the data from our previous study (*n* = 33) using identical analyses. The results reveal an opposite pattern from our previous study: participants in the neutral control condition showed poorer sleep (longer sleep onset latency, reduced slow-wave sleep, and lower SWA/beta ratio) compared to those anticipating stress. In a direct comparison of both studies, sleep parameters in the stress conditions were highly similar across the two studies, suggesting that the divergent outcomes are driven by differences in the control conditions. The temporal dynamic changes observed in our previous study could not be replicated. These findings highlight the importance of carefully considering control conditions in experimental sleep research and suggest that even “neutral” instructions can evoke anticipatory effects. Moreover, the observed benefits of anticipating post-sleep relaxation highlight opportunities for relaxation-based interventions to improve sleep quality.

## 1. Introduction

Psychosocial stress induces stress responses at both physiological and cognitive levels [1,2], subsequently disrupting sleep (e.g., [3,4]). Research findings have demonstrated that inducing psychosocial stress before sleep leads to prolonged sleep onset latency (SOL), greater sleep fragmentation, fewer deep sleep stages, and a decrease in objective measures of sleep quality (i.e., low-/high-frequency power ratio in electroencephalography (EEG) during nonrapid eye movement (NREM) sleep [4,5,6]). Most of these changes are typically observed in the early sleep period, with the late sleep period typically remaining unaffected. In a recently conducted study by our group, our results showed that anticipating a stressful situation after sleep primarily affected later sleep periods [7]. As one possible interpretation for this finding, we propose that cognitive arousal related to the upcoming event might either remain continuously active during sleep or is being reactivated toward the end of the sleep period in anticipation of the upcoming stressor. Such activation of cognitive arousal could, in turn, induce observed changes in physiological indicators during the late sleep period (e.g., changes in sleep parameters, heart rate, hormonal measures, etc.). The findings of our previous work support this notion, showing that activating the semantic concept of “relaxation” during NREM sleep promotes deeper sleep and lower cardiac activity [8,9]. By extension, we hypothesize that activating wake-related cognitions before sleep could elicit corresponding physiological responses associated with increased arousal and reduced sleep depth.

**Figure 1:**
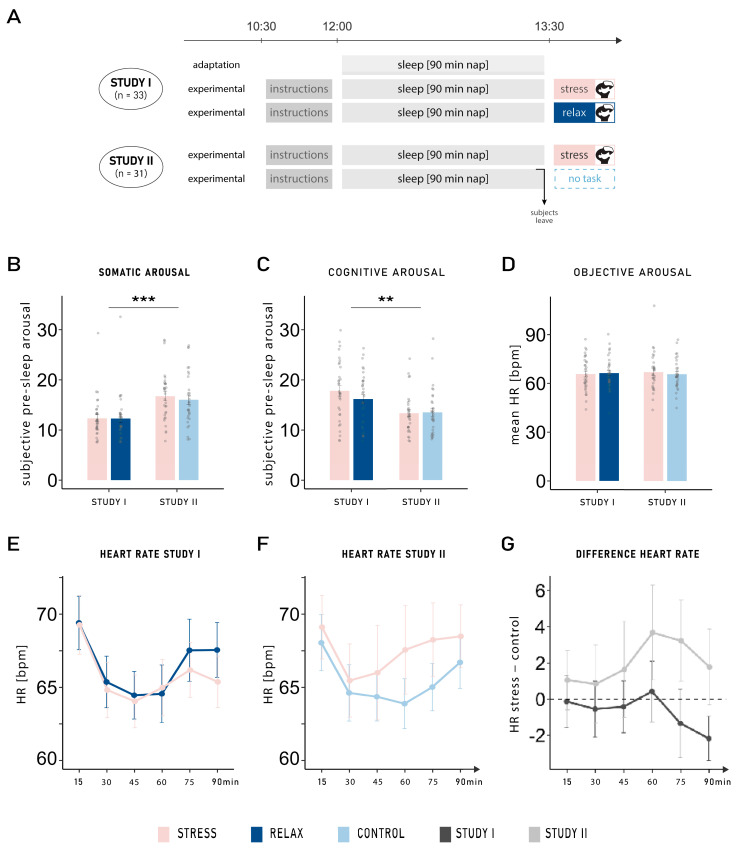
(**A**) Experimental procedure. In Study I ([7]), participants either anticipated a stress or a relaxation task to occur after sleep (*n* = 33). In Study II, the relaxation task was replaced by a neutral control condition (*n* = 31). The anticipated stress condition was the same in both studies: a virtual reality version of the TSST. In the relaxation task, subjects were told to relax in a beautiful virtual reality environment; in comparison, in the neutral control condition, they were informed before sleep that no further task would be conducted upon awakening. While participants slept for an adaptation nap before both experimental naps in Study I, no adaptation nap was performed in Study II. (**B**) Subjective pre-sleep arousal: Participants reported higher somatic pre-sleep arousal in Study II compared with Study I (main effect of study: *F*_1,62_ = 14.64, *p* < 0.001, *η*^2^ = 0.16), whereas the opposite result was found for (**C**) cognitive pre-sleep arousal (main effect of study: *F*_1,62_ = 10.31, *p* = 0.002, *η*^2^ = 0.12). (**D**) Mean heart rate (HR) in beats per minute (bpm) during the 90 min nap: No difference was observed between either group or condition (all *p*-values > 0.390). Progression of the mean heart rate across the nap period (15 min segments) in (**E**) Study I and (**F**) Study II, in the stress and control conditions. (**G**) Progression of changes in heart rate (HR) from the stress to the control condition: Heart rate was comparable between both conditions across all timepoints for both studies (*p* = 0.280). Means ± standard errors of the mean are displayed. ***: *p* ≤ 0.001; **: *p* ≤ 0.01.

The results indicating that anticipation of a stressful situation mainly affected later sleep periods were obtained by comparing the anticipation of a stressful task (Trier Social Stress Test; TSST [10]) to the anticipation of a relaxation task, both conducted in a virtual reality (VR) setting. It is thus possible that the detrimental effects on sleep observed in the anticipated stress condition may have been overestimated; since the anticipated relaxation task may have improved sleep parameters, it may have increased the contrast between conditions, thereby magnifying the effect of anticipated stress. To address this issue, we tested an additional group of participants who completed two nap sessions in our sleep laboratory. Under one condition, they underwent the exact same procedure of anticipated stress as in our previous study [7]; in comparison, under the other condition, they were explicitly told before sleep that the session would end directly after waking up. The control condition—and the omission of an adaptation nap—were the only differences in the procedure compared with the anticipation group in the study by Beck et al. ([7]; Study I: anticipated stress vs. relaxation; see Figure 1A). The newly acquired data set (Study II: anticipated stress vs. neutral control) was compared with the data from Beck et al.’s study [7], using the same analyses.

In the present study, we aimed to replicate and extend our previous study, in which we compared the anticipation of a stressful event with the anticipation of relaxation. Specifically, we sought to determine whether the previously observed effects of anticipated stress on sleep were influenced by the potentially beneficial effects of anticipating relaxation. To this end, we replicated the anticipated stress condition from our earlier work and added a neutral control condition as a comparison. In addition, we directly compared this new dataset to our previous study, to further investigate the temporal patterns of the influence of cognitive processes on sleep. To achieve these aims, 32 French- or German-speaking female subjects completed both sessions of the experiment. One subject was excluded due to technical problems during one session. The remaining 31 young female participants were compared to the 33 young female participants from Study I, resulting in a final sample of 64 individuals (mean age: 21.95 ± 2.46 [M ± SD]; age range: 18–30 years).

## 2. Results

### 2.1. Subjective and Objective Pre-Sleep Arousal

Subjective pre-sleep somatic arousal was significantly higher in Study II (16.39 ± 0.65) compared with Study I (12.30 ± 0.53; main effect of study: *F*_1,62_ = 14.64, *p* < 0.001, *η*^2^ = 0.16; see Figure 1B). No main effect of condition nor interaction was observed (both *p*-values > 0.450). However, the opposite result was found for subjective pre-sleep cognitive arousal, whereby higher cognitive arousal was found in Study I (17.00 ± 0.69) compared with Study II (13.42 ± 0.54; main effect of study: *F*_1,62_ = 10.31, *p* = 0.002, *η*^2^ = 0.12; see Figure 1C). No main effect of condition nor interaction was observed (both *p*-values > 0.117) and no difference was observed between both studies and both conditions (all *p*-values > 0.390) in objective arousal measured with the mean heart rate over the entire nap period (see Figure 1D). We further analyzed the progression of heart rate across the nap period in segments of 15 min. Heart rate was comparable between both conditions across all timepoints for both studies (interaction between condition, time, and study: *F*_3.61,223.93_ = 1.28, *p* = 0.280, *η*^2^ < 0.01; see Figure 1E–G).

### 2.2. Sleep Architecture

We observed general differences between Study I and Study II, independent of the experimental conditions. The main effect of study indicates that participants overall spent more time in sleep stage N3 in Study I (17.39 ± 1.81 min) compared with Study II (11.60 ± 1.42 min; *F*_1,62_ = 4.61, *p* = 0.036, η^2^ = 0.05), and less time in sleep stage N2 (Study I: 27.50 ± 1.51 min; Study II: 34.58 ± 1.40 min; *F*_1,62_ = 8.96, *p* = 0.004, η^2^ = 0.09). Furthermore, time spent in N1 was higher in Study I (12.20 ± 1.06 min) compared with Study II (7.45 ± 0.57 min; *F*_1,62_ = 9.90, *p* = 0.003, η^2^ = 0.11; see Table 1 for percentage values). These differences between studies remained when we restricted the analyses to the second experimental sessions (all *p*-values < 0.038), excluding the possibility that they are due to the lack of adaptation in Study II.

We observed two main effects of condition that reached the level of a statistical trend. In both studies, objectively measured time awake (wake after sleep onset; WASO) increased in the stress condition (6.53 ± 1.10 min) compared with the relaxation/control conditions (4.01 ± 0.73 min; *F*_1,62_ = 3.81, *p* = 0.055, *η*^2^ = 0.03). In addition, participants tended to show a reduced percentage of N2 sleep in the stress condition (41.26 ± 2.10%) compared with the relaxation/control conditions (45.15 ± 2.27%; *F*_1,62_ = 3.55, *p* = 0.064, *η*^2^ = 0.01). Subjective sleep quality and subjective sleep onset latency (SOL) were not affected by the conditions as no significant results were observed in either study (all *p*-values > 0.096).

**Figure 2:**
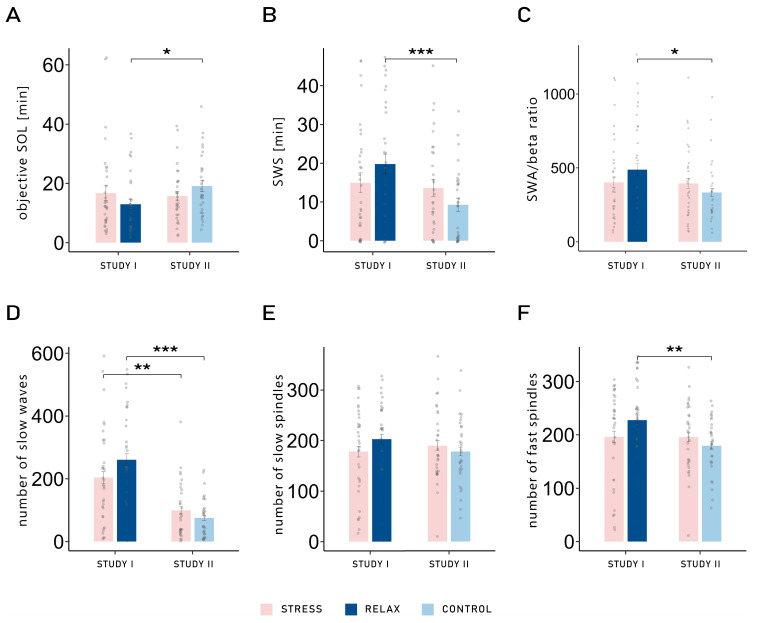
(**A**) Objective sleep onset latency: sleep onset latency was significantly longer in the neutral control condition of Study I compared with the relaxation condition of Study II (*t*_61.27_ = −2.43, *p* = 0.018, *d* = 0.608). This difference was not found when comparing both stress conditions (*p* = 0.746). (**B**) Time spent in slow-wave sleep (SWS) in minutes: Comparing the two control conditions, participants of Study I spent more time in SWS in the relaxation condition compared with participants of Study II in the neutral control condition (*t*_55.12_ = 3.38, *p* = 0.001, *d* = 0.833). This difference was not observed when comparing both stress conditions (*p* = 0.727). (**C**) Ratio between slow wave activity (SWA) and beta activity during NREM sleep, in the frontal area: The relaxation condition of Study I led to a significantly higher SWA/beta power ratio compared with the neutral control condition of Study II (*t*_53.31_ = 2.21, *p* = 0.031, *d* = 0.554). No difference was found when comparing the stress conditions of both studies (*p* = 0.925). (**D**) Number of slow waves (SWs) in the frontal area: More SWs were observed in Study I compared with Study II. However, the difference was bigger between both control conditions (*t*_43.04_ = 6.32, *p* < 0.001, *d* = 1.544), compared with the difference between both stress conditions (*t*_51.73_ = 3.34, *p* = 0.002, *d* = 0.823). A similar pattern was found for (**E**) the number of slow spindles in the frontal area; however, the differences between conditions across studies were not significant (both *p*-values > 0.185). (**F**) Number of fast spindles in the parietal area: The control conditions differed significantly, with more fast spindles observed in the relaxation condition of Study I compared with the neutral control condition of Study II (*t*_53.88_ = 2.79, *p* = 0.007, *d* = 0.688). However, the stress conditions did not differ across studies (*p* = 0.961). Means ± standard errors of the mean are displayed. ***: *p* ≤ 0.001; **: *p* ≤ 0.01; *: *p* ≤ 0.05.

We also observed several significant interactions between the factors study and condition, all of which followed the same pattern. For example, a significant interaction was observed for objective SOL (*F*_1,62_ = 7.71, *p* = 0.007, *η*^2^ = 0.03). Post hoc pairwise *t*-tests revealed that the SOL was significantly shorter in the relaxation condition of Study I (12.94 ± 1.74) compared with the neutral control condition of Study II (19.15 ± 1.88; *t*_61.27_ = −2.43, *p* = 0.018, *d* = 0.608). This difference was not found when comparing both stress conditions (Study I: 16.76 ± 2.57; Study II: 15.76 ± 1.67; *p* = 0.746; see Figure 2A). No main effect was observed (both *p*-values > 0.308). The same interaction was also observed for the amount of SWS (minutes: *F*_1,62_ = 6.43, *p* = 0.014, *η*^2^ = 0.03; percentage: *F*_1,62_ = 5.68, *p* = 0.020, *η*^2^ = 0.03): comparing the two control conditions, participants of Study I spent more time in SWS in the relaxation condition (19.80 ± 2.56 min) compared with participants of Study II in the neutral control (9.40 ± 1.71 min; *t*_55.12_ = 3.38, *p* = 0.001, *d* = 0.833). Again, this difference was not observed when comparing both stress conditions (Study I: 14.97 ± 2.52; Study II: 13.79 ± 2.23; *p* = 0.727; see Figure 2B and Table 1). No other effect was observed in objective sleep variables (all *p*-values > 0.110; see Table 1).

Objective sleep quality was also measured with the SWA/beta power ratio in NREM sleep over the frontal electrodes. Two participants were excluded from this analysis due to values larger than 3 SD above the mean. A significant interaction was once again observed between study and condition (*F*_1,60_ = 4.07, *p* = 0.048, *η*^2^ = 0.02), demonstrating the same pattern. The relaxation condition of Study I led to a significantly higher SWA/beta ratio (487.45 ± 40.61) compared with the neutral control condition of Study II (395.81 ± 33.63; *t*_53.31_ = 2.21, *p* = 0.031, *d* = 0.554). No difference was observed when comparing the stress conditions (Study I: 402.36 ± 35.64; Study II: 395.81 ± 33.63; *t*_59.93_ = 0.09, *p* = 0.925, *d* = 0.024; see Figure 2C and Table 2). Therefore, while both stress conditions exhibited similar sleep parameters, differences were observed in the control conditions: the relaxation condition led to objectively improved sleep quality (decreased SOL, more time spent in SWS, and increased SWA/beta power ratio) compared with the neutral control condition. Comparisons between conditions (stress vs. relaxation/control) within studies are presented in Supplementary Table S1.

### 2.3. Slow Waves and Sleep Spindles

We performed further analyses on different characteristics of single slow waves (SWs) and slow spindles in frontal electrodes and fast spindles in parietal electrodes (count, density, duration, amplitude, and up- and down-slope). Firstly, it is important to note that in general, there were more SWs in Study I (232.38 ± 19.25) compared with Study II (87.36 ± 9.88) and that those SWs showed an increased density, amplitude, and down- and up-slope in Study I compared with Study II (all *p*-values for the main effect of study < 0.001). However, SWs were of longer duration in Study II (1.72 ± 0.01) compared with Study I (1.25 ± 0.01; main effect of study: *F*_1,62_ = 1145.13, *p* < 0.001, *η*^2^ = 0.94). Similarly, participants of Study I showed an increased density (*F*_1,62_ = 9.04, *p* = 0.004, *η*^2^ = 0.11) and amplitude (*F*_1,62_ = 24.48, *p* < 0.001, *η*^2^ = 0.27) of fast spindles; however, they tended to be of shorter duration (*F*_1,62_ = 3.27, *p* = 0.075, *η*^2^ = 0.04) compared with Study II. Following the pattern observed in sleep parameters, the interaction between study and condition was significant for the number of SWs (*F*_1,62_ = 7.92, *p* = 0.007, *η*^2^ = 0.03; see Figure 2D) and fast spindles (*F*_1,62_ = 5.76, *p* = 0.019, *η*^2^ = 0.03; see Figure 2F) and showed a statistical trend for the number of slow spindles (*F*_1,62_ = 3.42, *p* = 0.069, *η*^2^ = 0.01; see Figure 2E). Regarding the number of SWs, the difference in SWs between the relaxation and neutral control conditions was stronger (Study I: 260.82 ± 19.00; Study II: 75.42; *t*_43.04_ = 6.32, *p* < 0.001, *d* = 1.544) compared with the difference in SWs between both stress conditions (Study I: 203.94 ± 18.90; Study II: 99.31 ± 11.25; *t*_51.73_ = 3.34, *p* = 0.002, *d* = 0.823). The same pattern was observed for SW density (interaction: *F*_1,62_ = 5.47, *p* = 0.023, *η*^2^ = 0.02) and their duration (interaction: *F*_1,62_ = 4.73, *p* = 0.033, *η*^2^ = 0.01). While the post hoc pairwise *t*-tests were not significant in terms of the number of slow spindles (both *p*-values > 0.185), the fast spindles exhibited the same pattern as observed with the other variables. Indeed, the relaxation and neutral control conditions differed significantly, with more fast spindles observed in the relaxation conditions of Study I (227.27 ± 10.09) compared with the neutral control condition of Study II (179.84 ± 6.65; *t*_53.88_ = 2.79, *p* = 0.007, *d* = 0.688). However, the stress conditions did not differ across studies (Study I: 196.27 ± 10.69; Study II: 195.32 ± 8.32; *p* = 0.961). The main effect of condition was significant only for the duration of SWs, with longer SWs observed in the control conditions (1.48 ± 0.03) compared with the stress conditions (1.46 ± 0.03; *F*_1,62_ = 4.01, *p* = 0.050, *η*^2^ = 0.01), and showed a statistical trend for the down-slope of SWs, with bigger slopes in the control conditions (−250.18 ± 20.66) compared with the stress conditions (−238.18 ± 18.66; *F*_1,62_ = 3.52, *p* = 0.065, *η*^2^ < 0.01). No other differences were observed (all *p*-values > 0.125; see Table 2 for all values).

### 2.4. Dynamic Changes of SWA/Beta Power Ratio, Slow Waves, and Sleep Spindles

**Figure 3:**
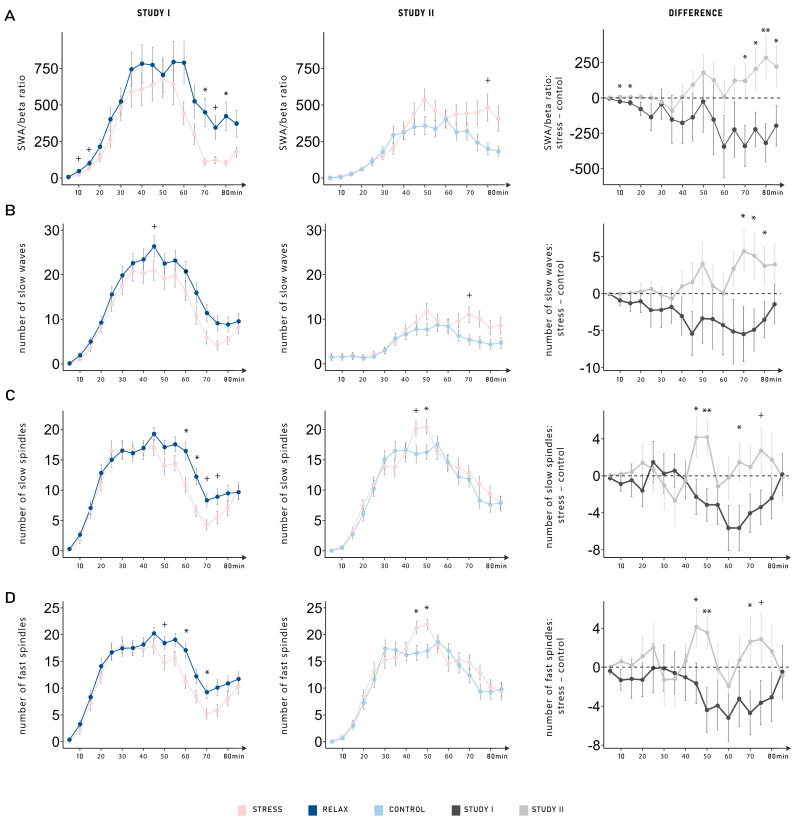
Dynamic changes in (**A**) the ratio between slow-wave activity (SWA) and beta activity, (**B**) the number of slow waves, (**C**) the number of slow spindles, and (**D**) the number of fast spindles. Among all variables, a consistent pattern was observed: the different conditions (stress vs. relaxation/control) had distinct impacts on sleep in each study; however, there was no significant dynamic change across the nap period (condition, study interactions; all *p*-values < 0.045). Data is shown as the means ± standard errors of the mean averaged over 5 min segments for all conditions (stress, relaxation, and control). The left-hand column displays the data from Study I (anticipated stress vs. relaxation). The middle column displays the data from Study II (anticipated stress vs. neutral control). In the right-hand column, differences between the stress and the control (relaxation or neutral control) conditions are shown separately for Study I (dark gray) and Study II (light gray). **: *p* ≤ 0.01; *: *p* ≤ 0.05; ^+^: *p* ≤ 0.08.

The results almost uniformly indicate that anticipating a relaxation task after sleep seems to increase objective sleep quality. In Study I (anticipated stress vs. relaxation), the results showed that the anticipation of a task specifically impacted the late part of the nap [7]. Therefore, we further explored whether there were dynamic changes in SWA/beta power ratio, slow waves and spindle parameters (number, density, duration, and amplitude). We explored the progression of each variable, focusing on differences between conditions across both studies (see Figure 3A–D). Two participants were excluded from the analysis of the SWA/beta power ratio due to values larger than 3 SD of the mean in all electrodes. Among all variables, we observed a consistent pattern: the different conditions (stress vs. relaxation/neutral control) had distinct impacts on sleep in each study; however, there was no dynamic change across the nap period. This finding was supported by significant interactions between the factors condition (stress vs. relaxation/control) and study (Study I vs. Study II) in all variables (all *p*-values < 0.045; see Table 2) except for the amplitude of slow spindles and the duration of fast spindles, for which it showed a statistical trend (both *p*-values < 0.078). For all variables, the three-way interaction (condition–study–time) was not significant (all *p*-values > 0.195).

## 3. Discussion

By introducing a new group, we aimed to replicate and extend the findings of Beck et al. [7]. More specifically, we aimed to clarify whether the disparity between the stress and relaxation conditions observed in our initial study could be attributed to a contrast effect (see [12]) stemming from the anticipation of relaxation.

Interestingly, our results reveal an opposite pattern: participants exhibited worse sleep parameters (SOL, amount of SWS, and SWA/beta ratio) in the neutral condition compared with the anticipated stress condition. When directly comparing both studies, sleep parameters in the stress conditions was highly similar across the two studies, suggesting that the divergent outcomes are driven by differences in the control conditions: whereas anticipating relaxation appeared to promote sleep in Study I (anticipated stress vs. relaxation [7]), the neutral control in Study II (anticipated stress vs. neutral control) was associated with poorer sleep quality. Previous studies on the effects of anticipated stress on sleep have shown mixed results: for example, Elder et al. [13] observed no difference in objective and subjective sleep when subjects were anticipating cognitive demands the following day, in comparison to when they were expecting the following day to include only sedentary activities. In another study, in which the anticipation of two different levels of stress (high vs. low) was compared, the authors demonstrated discrepancies in sleep architecture between both levels, with no differences being identified in comparison to a neutral control condition [14]. This finding suggests that it may not be the anticipation of stress alone that disrupts sleep, but rather that the nature and valence of what is anticipated play a critical role in modulating pre-sleep cognitive and emotional states, subsequently shaping sleep architecture.

In our previous study, we reported the largest differences in sleep parameters (SWA/beta ratio, SWs, and spindles) towards the end of the nap period, closer to the anticipated stressor [7]. This finding could not be replicated in our comparison with a neutral control condition, as no dynamic changes were observed. Sleep parameters appeared to be generally better across the entire nap period in the relaxation condition compared with the neutral control. However, the decrease in sleep parameters towards the end of the nap in anticipation of the stressor observed in Study I was not replicated in Study II. Overall, participants exhibited worse sleep parameters in Study II than in Study I (e.g., less SWS and fewer SWs), despite the similarity in procedure (same environment and same instructions). This finding underscores the importance of within-subjects design in sleep research, as between-group differences may reflect individual variability, with sleep dynamics remaining consistent within each study group. Importantly, this global difference does not account for the reversal in the pattern of condition effects. Specifically, the strongest contrast arises from the relaxation versus neutral control comparison, suggesting that the anticipation of relaxation exerts a measurable benefit on sleep. By contrast, while subtle differences in the progression of sleep were observed between the two stress conditions (Study I vs. Study II), their overall sleep architecture was not significantly different.

From the above findings, it can be concluded that while participants from Study II slept less deeply overall, this factor alone cannot explain why the conditions led to an opposite pattern to the results observed in Study I. Differences in subjective pre-sleep arousal across studies provide a more plausible explanation: subjective somatic arousal was higher in Study II, while cognitive arousal was lower, regardless of conditions (stress or relaxation/control). These differences may reflect variations in how participants appraised the experimental context or perceived control over outcomes, aligning with literature sources suggesting that anticipatory appraisals influence both arousal and sleep (e.g., [15,16]). The absence of heart rate differences suggests that subtle physiological changes accompany these anticipatory effects, even if they do not reach statistical significance.

It is important to note that, unlike in Study I, no adaptation nap was implemented in Study II. Although we attempted to control for potential confounding factors arising from this factor in our analyses, we cannot fully exclude the possibility that the lack of an adaptation nap influenced the observed results. A further point of caution concerns the distribution of chronotypes across the two datasets. All participants in both studies fell within the moderate chronotype range (i.e., moderately morning, neutral, moderately evening), with no extreme morning- or evening-type individuals. However, we observed slight differences across studies which may have contributed, at least in part, to the variability observed between them and should therefore be considered when interpreting cross-study comparisons. Moreover, as the present study included only female participants, potential influences of the menstrual cycle on sleep parameters should be considered. Although findings in the literature are inconsistent, studies in healthy young women generally report no major changes in objective sleep measures (e.g., SOL, sleep efficiency, SWS, or SWA) across the cycle, despite some variations in subjective sleep quality. The main cycle-related differences are typically observed in REM sleep, stage N2, and spindle activity [17,18,19]. Thus, while menstrual cycle effects are likely minor overall, caution is warranted when interpreting our results, specifically on spindle activity. 

To conclude, these findings highlight the importance of carefully considering control conditions in experimental sleep research, as “neutral” instructions may also carry anticipatory implications, significantly affecting sleep. Moreover, the observed benefits of anticipating post-sleep relaxation reinforce the potential of relaxation-based interventions. Relaxation techniques have already been shown to improve sleep quality by reducing both physiological and cognitive pre-sleep arousal (e.g., [20,21,22]). In previous studies, we posited that pre-sleep cognitive content may be spontaneously reactivated during subsequent sleep [23,24], similarly to the reactivation of memories during sleep (e.g., [25,26]). Such reactivations may in turn trigger associated physiological responses, thereby modulating sleep quality. Supporting this framework, presenting relaxation-related word cues during sleep has been shown to increase time spent in SWS, enhance slow oscillatory power and slow cardiac activity [8,9]. Moreover, anticipating a post-sleep relaxation may have shaped appraisal and subjective benefit, similarly to expectancy effects observed in placebo research [27,28]. On the other hand, a neutral control condition may have elicited adverse feelings such as boredom or impacted mood due to speculations on the purpose of this condition. Together, these findings suggest that relaxation-oriented pre-sleep cognitive activity may improve sleep by engaging sleep-promoting neural networks through spontaneous reactivations during sleep.

## 4. Materials and Methods

### 4.1. Participants

A newly acquired data set (Study II) was compared with the data from the work of Beck et al. ([7]; Study I). In Study II, thirty-two healthy French- or German-speaking female subjects completed both sessions of the experiment. One subject was excluded due to technical problems during one session. The remaining 31 young female participants (mean age: 21.58 ± 1.91 [M ± SD]) were compared to the 33 young female participants from Study I (mean age: 22.30 ± 2.87 [M ± SD]), resulting in a final sample of 64 individuals (mean age: 21.95 ± 2.46 [M ± SD]; age range: 18–30 years). The data set from our previous study [7] was collected from August 2019 to March 2020, and the new data set was collected from October 2023 to June 2024. Given the substantial overlap between these periods, potential seasonal effects, such as variations in natural light exposure, are unlikely to have had a major influence on the results. All participants of both studies were of moderate chronotypes (i.e., moderately morning, neutral, moderately evening), with no extreme morning- or evening-type individuals. However, the distribution of moderate chronotypes differed significantly between the datasets (Fisher’s Exact Test, *p* = 0.047): Study I included more moderately evening-type participants (30%), whereas Study II comprised a higher proportion of neutral-type participants (84%). This difference should be considered when interpreting cross-study comparisons, although the main pattern of results is unlikely to be fully explained by chronotype differences.

Participants were recruited via announcements posted at the University of Fribourg and shared on social media platforms. In return for their participation, they received course credits. Eligibility criteria included the absence of medication influencing sleep, as well as no history of neurological, psychiatric or sleep-related disorders. Individuals working night shift or who had travelled across time zones within the 6 weeks preceding the experiment were excluded. Participants were asked to refrain from consuming alcohol or caffeinated drinks on the day before and during the experimental sessions. The study protocol was approved by the Internal Review Board of the Department of Psychology at the University of Fribourg (approval No. 475). All participants provided written informed consent prior to the study, which was conducted in accordance with the principles of the Declaration of Helsinki.

### 4.2. Design and Procedure

Each participant participated in two experimental sessions including a 90 min nap in the sleep laboratory of the University of Fribourg. In one session, a stressful task was performed post-nap in a virtual reality environment, whereas in the other session, either a relaxation task in a virtual reality environment (Study I) or no task (Study II) was performed post-nap. The order of the conditions (stress or relaxation/control) was counterbalanced across participants according to a within-subject design. Only participants from Study I completed an adaptation nap one week prior to the first experimental nap. In both studies, no sleep deprivation was applied before testing and participants adhered to their habitual sleep–wake rhythm.

Both experimental sessions started between 10:30 and 11:00 a.m. and took place on the same day of the week, one week apart (except for one participant who had a four-week gap between both sessions due to illness). Both sessions started with the attachment of electrodes for recordings of electroencephalography (EEG), electromyography (EMG), electrooculography (EOG), and electrocardiography (ECG) data. Thereafter, participants received the instructions for the post-nap procedure (stressful task or relaxation/no task) and then went to bed for a 90 min sleep opportunity. After 90 min, participants were woken up, and their subjective sleep quality and mood were assessed. Upon awakening, participants either performed the stressful task or the relaxation task/no task (see Figure 1A for a summary of the experimental procedure). Participants completed standardized questionnaires (see Questionnaires) throughout the sessions.

### 4.3. Stressful Tasks in Virtual Reality

The task and material used in the present manuscript are the same as the one described in our previous study (see [7] for further information on the procedure and material used). The stress and relaxation tasks were conducted using an HTC VIVE PRO head -mounted display (https://www.vive.com, accessed on the 6 October 2025).

To induce acute social stress, a virtual reality version of the Trier Social Stress Test (TSST [10]) was used in the stress condition. The TSST is an established method to induce acute social stress in a laboratory setting [29] and induces a similar stress response when conducted in virtual reality [30,31]. In our studies, the TSST comprised two consecutive parts: a 5 min speech followed by a 5 min mental arithmetic task, both performed while standing in front of a panel of three university professors. In the first part, participants were instructed to deliver a persuasive 5 min speech explaining why they were the ideal candidate for their “dream job”. If they stop talking before the end of the allotted 5 min, standardized questions were asked in accordance with the TSST manual. Immediately afterward, participants were required to count backward from 2023 in steps of 17 for 5 min in front of the same jury panel. Whenever an error was made, they were instructed to restart the task from 2023. The same 15 min pre-recorded virtual reality video was used as in our previous study [7] and displayed using Skybox VR Player (https://skybox.xyz/en/, accessed on the 6 October 2025). Before sleep and again before performing the task, participants viewed on-screen instructions informing them that (1) their performance would be recorded via video and audio for subsequent behavioral and vocal analyses; (2) the task is scientifically recognized to elicit both psychological and physiological stress responses; (3) high performance on this task reflects strong stress resilience, whereas lower performance is associated with elevated risk for conditions such as cardiovascular disease, sleep problems, depression, and burnout; (4) results would be compared to peers of a similar age group and participants demonstrating excellent performance in both the speech and arithmetic segments would receive an additional compensation of 10 CHF; and (5) their contribution was essential for the success of the research project associated dissertations. Following the task, participants were verbally debriefed and informed that no actual recordings had been made and that all participants would receive the 10 CHF bonus regardless of performance. They subsequently signed a written debriefing form confirming this information at the end of the experiment. Participants received the instructions for the task before sleep and again, after sleep, before conducting the task. Finally, participants rated their subjective stress level at three time points (before the task began, after the speech, and following the arithmetic task) using a 10-point Likert scale (“To what extent do you feel stressed right now on a scale from 1 (=not at all) to 10 (=extremely)?”).

In the neutral control condition (Study II), participants received the instructions before the nap that no task was to be performed upon awakening, and they would only be required to complete questionnaires. The procedure for the relaxation task is described in the work of Beck et al. [7].

### 4.4. Questionnaires

The same questionnaires were used as in our previous study [7]. Questionnaires completed after sleep included the Presleep Arousal Scale (PSAS [32]) and a subjective sleep quality questionnaire (SF-A/R [33]). Before and after sleep in both sessions, the level of stress was assessed with a single question: “To what extent do you feel stressed right now on a scale from 1 (=not at all) to 10 (=extremely)?”. This question was asked again at the beginning, after 5 min and at the end of the stressful task.

Immediately upon awakening, participants completed the PSAS [32] to evaluate both cognitive and somatic components of pre-sleep arousal. The scale comprises 16 items rated on a 5-point Likert scale ranging from 1 (=not at all) to 5 (=extremely), reflecting the intensity of symptoms experienced before sleep onset. Eight items assess cognitive arousal (e.g., worrying about sleep, rumination, and anxious or depressive thoughts) while the remaining eight capture somatic arousal (e.g., sensations of a racing heart or muscle tension). Separate subscale scores are computed for cognitive and somatic arousal, each ranging from 8 to 40, with higher values indicating greater arousal intensity. A cognitive arousal score exceeding 16 is considered indicative of elevated pre-sleep activation [15].

Subjective sleep quality was assessed in the morning using the sleep quality subscale of the SF-A/R [33]. This measure includes four indices addressing difficulties with sleep initiation and maintenance, premature awakening with inability to resume sleep, and general characteristics of sleep quality. Each item is rated from 1 (absence of good sleep characteristics) to 5 (strong presence of good sleep characteristics). Additionally, subjective sleep onset latency (SOL) was rated as an indicator of perceived time to fall asleep.

### 4.5. EEG Recording

Electroencephalographic (EEG) signals were recorded using 12 gold cup electrodes placed according to the international 10–20 system (C3, C4, Cz, F3, F4, Fpz, M1, M2, P3, P4, O1, and O2). Recordings were acquired with a BrainAmp amplifier (Brain Products, Gilching, Germany), at a sampling rate of 500 Hz. The Cz electrode served as the physical reference, and Fpz was used as the ground. Additional electrodes were applied to record electrooculographic (EOG) activity (two electrodes), electromyographic (EMG) activity from the chin (two bipolar electrodes), and electrocardiographic (ECG) activity (two electrodes). Electrode impedances were maintained below 10 kΩ. During the experimental tasks, two additional electrodes were affixed to the participant’s left palm to measure electrodermal activity (EDA).

In accordance with the AASM recommendations [34], EEG electrodes were re-referenced offline to the contralateral mastoids for analysis and sleep staging. Preprocessing was conducted with BrainVision Analyzer 2.2 (Brain Products, Gilching, Germany), using standard AASM filtering parameters (e.g., EEG 0.3–35 Hz). Sleep stages were visually scored offline by an independent rater following standard AASM criteria [34]. To complement manual scoring, an automated online algorithm (U-Sleep; https://sleep.ai.ku.dk/ accessed on the 6 October 2025 [35]) was also employed. In the event of discrepancy between manual and automated classifications, a third expert scorer provided the final decision.

### 4.6. Preprocessing and Artifact Rejection

EEG preprocessing was conducted using BrainVision Analyzer 2.2 (Brain Products, Gilching, Germany). The continuous data was filtered with a 0.1 Hz high-pass and 40 Hz low-pass filter, along with a 50 Hz notch filter to remove line noise. Signals were re-referenced to the average of the mastoid electrodes. Based on sleep stage scoring, NREM sleep periods were segmented into 30 s epochs and subsequently divided into segments of 2048 data points (4 s, 102 points overlap). Artifact rejection was performed automatically according to the following criteria: (1) EMG activity difference below 150 µV across both EMG channels, (2) voltage steps not exceeding 50 µV/ms in any EEG channel, (3) maximum EEG amplitude difference below 500 µV, and (4) a minimum EEG signal amplitude greater than 0.5 µV across all channels. These automatic rejection parameters have been validated in previous studies from our group (e.g., [7,23]). The number of excluded segments was subsequently verified by manual inspection.

### 4.7. Power Analysis and Slow Wave and Spindle Detection

For the analyses of oscillatory activity during sleep (power analysis and spindle and slow wave detection), artifact-rejected data was exported as continuous data and further analyzed with the SleepTrip toolbox (https://github.com/Frederik-D-Weber/sleeptrip, accessed on the 6 October 2025) for Matlab R2024b (Mathworks, Natick, MA, USA) which is based on FieldTrip functions [36]. We used the default settings of SleepTrip (10% segment overlap, 20% Hanning window) to calculate the average power values (µV2) during NREM sleep for the slow wave activity (SWA; 0.5–4.5 Hz) and beta activity (15–30 Hz) for each channel (F3, F4, C3, C4, P3, P4, O1, and O2). We then computed the ratio between SWA and beta activity as this measure is associated with objective sleep quality [7,37]. Outliers were identified based on SWA values (criterion: 3 SD ± mean) and replaced with values from the contralateral electrode. One subject in each group was excluded from the power analysis due to SWA values exceeding the outlier criterion in all electrodes in one session. Additional exploratory analyses on 5 min segments of sleep were conducted on the sleep scoring data without the application of an artifact rejection procedure.

Following the procedure described by Beck et al. [7], slow wave characteristics during NREM sleep were quantified for each participant and EEG channel. The number of slow waves and their density per 30 s epoch of NREM sleep were determined, along with their mean amplitude and duration. Additionally, the descending (down-) slope was computed as the ratio between the negative half-wave peak and the time interval from the first zero-crossing to the trough (expressed in µV/s), whereas the ascending (up-) slope was defined as the ratio between the absolute value of the negative half-wave peak and the duration from the trough to the subsequent zero-crossing (also in µV/s). Similarly to our previous study [7], average slow spindle peaks for the newly acquired data set (Study II) ranged from 9.1 to 13.6 Hz with an average frequency of 11.48 ± 1.18 Hz (M ± SD), and fast spindle peaks ranged from 12.4 to 15.3 Hz with an average frequency of 14.07 ± 0.56 Hz (M ± SD).

### 4.8. Electrocardiography

The electrocardiogram (ECG) results were analyzed using Kubios HRV Premium 3.4.3 (Kubios Oy, Kuopio, Finland). We analyzed the ECG signal from the complete nap and from 15 min segments. The ECG data was exported in EDF+ format using BrainVision Analyzer 2.2.1.8266 (BrainProducts GmbH, Gilching, Germany). Kubios HRV Premium offers automatic artefact correction based on the RR series (the interval between two R-signals) to eliminate ectopic beats and artefacts in unfiltered data [38]. The data was then analyzed in the time and frequency domain and the mean heart rate (mean HR measured in beats per minute [bpm]) was used as an index for physiological arousal [1,7,23].

### 4.9. Statistical Analyses

Statistical analyses were performed using RStudio version 2025.09.1 (R Core Team). Data is presented as the means ± standard errors. To compare the effects of the anticipation of a stressful task, a relaxation task or no task on sleep, we performed a mixed-design analysis of variance (ANOVA) containing the within-subject factor condition (stress vs. relaxation/control) and the between-subject factor group (Study I vs. Study II). Post hoc tests for significant interactions and main effects consisted of uncorrected paired Student’s *t*-tests. In the event of statistically significant results, effect sizes are reported with partial eta squared (*η^2^*) for main effects and interactions and Cohen’s *d* for *t*-tests. Where the assumption of sphericity was violated, Greenhouse-Geisser corrected *p*-values are reported. The level of significance was set to *p* < 0.05.

### 4.10. Preregistration

The procedure for Study I was preregistered and can be viewed via the following link: https://osf.io/rf6tc (accessed on the 6 October 2025).

## Figures and Tables

**Table 1:** Subjective and objective sleep parameters in the stress and relaxation/neutral control conditions, in both studies.

	Study I Anticipated Stress vs. Relaxation		Study II Anticipated Stress vs. Neutral Control		Main Effect of Study		Main Effect of Condition		Interaction Effect	
	Stress	Control	Stress	Control	*p*	*η* ^2^	*p*	*η* ^2^	*p*	*η* ^2^
**Subjective Sleep Parameters**										
Cognitive PSA	17.82 ± 1.03	16.18 ± 0.91	13.32 ± 0.72	13.52 ± 0.83	**0.002**	**0.12**	0.214	<0.01	0.117	<0.01
Somatic PSA	12.33 ± 0.70	12.27 ± 0.80	16.74 ± 0.90	16.03 ± 0.94	**<0.001**	**0.16**	0.450	<0.01	0.524	<0.01
Sleep Quality	3.56 ± 0.14	3.57 ± 0.13	3.71 ± 0.12	3.65 ± 0.11	0.416	<0.01	0.785	<0.01	0.729	<0.01
SOL	23.18 ± 1.93	20.45 ± 1.97	25.16 ± 1.97	22.58 ± 1.70	0.352	<0.01	0.096	0.02	0.963	<0.01
**Objective Sleep Parameters**										
SOL [min]	16.76 ± 2.57	12.94 ± 1.74	15.76 ± 1.67	19.15 ± 1.88	0.308	0.01	0.869	<0.01	**0.007**	**0.03**
WASO [%]	9.93 ± 2.63	5.74 ± 1.93	8.10 ± 1.54	6.79 ± 1.86	0.857	<0.01	0.163	0.01	0.463	<0.01
N1 [%]	20.05 ± 2.75	17.07 ± 2.22	9.63 ± 1.13	11.30 ± 1.49	**0.003**	**0.11**	0.595	<0.01	0.064 ^a^	0.01
N2 [%]	37.82 ± 2.61	39.70 ± 2.57	44.92 ± 3.24	50.96 ± 3.55	**0.015**	**0.07**	0.064 ^a^	0.01	0.324	<0.01
SWS [%]	20.55 ± 3.14	26.23 ± 3.20	18.40 ± 3.37	12.21 ± 2.28	**0.024**	**0.05**	0.918	<0.01	**0.020**	**0.03**
REM [%]	11.64 ± 1.71	11.27 ± 1.77	12.55 ± 2.17	12.37 ± 2.54	0.656	<0.01	0.882	<0.01	0.959	<0.01
TST [min]	63.67 ± 3.48	68.30 ± 3.16	64.81 ± 3.00	61.68 ± 2.96	0.472	<0.01	0.754	<0.01	0.110	0.01
WASO [min]	7.18 ± 1.89	3.36 ± 0.85	5.84 ± 1.05	4.69 ± 1.20	0.996	<0.01	0.055 ^a^	0.03	0.297	<0.01
N1 [min]	13.14 ± 1.64	11.26 ± 1.35	7.35 ± 0.80	7.55 ± 0.82	**0.003**	**0.11**	0.335	<0.01	0.236	<0.01
N2 [min]	26.50 ± 2.21	28.50 ± 2.07	33.95 ± 2.37	35.21 ± 1.52	**0.004**	**0.09**	0.351	<0.01	0.831	<0.01
SWS [min]	14.97 ± 2.52	19.80 ± 2.56	13.79 ± 2.23	9.40 ± 1.71	**0.036**	**0.05**	0.903	<0.01	**0.014**	**0.03**
REM [min]	9.06 ± 1.38	8.74 ± 1.42	9.71 ± 1.70	9.45 ± 1.92	0.699	<0.01	0.844	<0.01	0.984	<0.01

Notes: Subjective parameters are based on subjective ratings in the PSAS, assessing pre-sleep arousal (PSA) and SF-A/R questionnaire, measuring subjective sleep quality and sleep onset latency (SOL). Objective values are based on polysomnographic recordings. Sleep stages N1, N2, slow-wave sleep (SWS), rapid-eye movement (REM) sleep, wake after sleep onset (WASO), SOL, SWS latency, REM sleep latency are measured in minutes, and percentages indicate partial percentage of total sleep time (TST). Values are means and standard errors of the mean. Significant results are highlighted in bold. ^a^ indicates *p* < 0.07. *η*^2^ represents the effect sizes where a value below or equal to 0.02 reflects a small effect, a value between 0.02 and 0.14 reflects a medium effect, and a value above 0.14 reflects a large effect.

**Table 2:** SWA/beta power ratio and characteristics of slow waves, slow spindles and fast spindles in NREM sleep, in both conditions and both studies. The last columns refer to the interaction between condition and study in the analysis of 5 min segments, adding the factor time.

	Study I Anticipated Stress vs. Relaxation		Study II Anticipated Stress vs. Neutral Control		Main Effect of Study		Main Effect of Condition		Interaction Effect		In 5-Min Segments: Interaction Effect	
	Stress	Control	Stress	Control	*p*	*η* ^2^	*p*	*η* ^2^	*p*	*η* ^2^	*p*	*η* ^2^
**SWA/beta ratio in frontal area**												
	402.36 ± 35.64	487.45 ± 40.61	395.81 ± 33.63	334.60 ± 27.12	0.187	0.02	0.743	<0.01	**0.048**	**0.02**	**0.018**	**0.01**
**Slow waves in frontal area**												
Count	203.94 ± 18.90	260.82 ± 19.00	99.31 ± 11.25	75.42 ± 8.09	**<0.001**	**0.26**	0.255	<0.01	**0.007**	**0.03**	**0.005**	**<0.01**
Amplitude	138.27 ± 4.30	144.79 ± 4.31	83.75 ± 2.19	83.14 ± 2.02	**<0.001**	**0.54**	0.237	<0.01	0.154	<0.01	**0.005**	**<0.01**
Density	2.29 ± 0.14	2.63 ± 0.14	1.02 ± 0.10	0.83 ± 0.07	**<0.001**	**0.42**	0.538	<0.01	**0.023**	**0.02**	**0.005**	**<0.01**
Duration	1.25 ± 0.01	1.25 ± 0.01	1.70 ± 0.01	1.73 ± 0.01	**<0.001**	**0.94**	**0.050**	**0.01**	**0.033**	**0.01**	**0.011**	**<0.01**
Up-slope	381.44 ± 14.44	386.66 ± 14.67	109.94 ± 2.98	109.31 ± 2.83	**<0.001**	**0.73**	0.771	<0.01	0.711	<0.01	**0.027**	**<0.01**
Down-slope	−365.17 ± 11.88	−388.86 ± 13.86	−102.99 ± 2.97	−102.55 ± 2.76	**<0.001**	**0.77**	0.065 ^a^	<0.01	0.056 ^a^	<0.01	**0.011**	**<0.01**
**Slow spindles in frontal area**												
Count	178.36 ± 10.70	202.70 ± 9.68	190.00 ± 9.48	178.21 ± 8.55	0.703	<0.01	0.523	<0.01	0.069 ^a^	0.01	**0.034**	**<0.01**
Amplitude	30.56 ± 0.73	30.77 ± 0.86	28.44 ± 0.87	29.93 ± 0.99	0.349	0.01	0.210	<0.01	0.343	<0.01	0.078 ^a^	<0.01
Density	2.25 ± 0.06	2.19 ± 0.05	2.17 ± 0.08	2.16 ± 0.06	0.647	<0.01	0.518	<0.01	0.600	<0.01	**0.023**	**<0.01**
Duration	0.87 ± 0.01	0.86 ± 0.01	0.84 ± 0.01	0.85 ± 0.01	0.284	0.02	0.845	<0.01	0.295	<0.01	**0.048**	**<0.01**
**Fast spindles in parietal area**												
Count	196.27 ± 10.69	227.27 ± 10.09	195.32 ± 8.32	179.84 ± 6.65	0.125	0.03	0.426	<0.01	**0.019**	**0.03**	**0.022**	**<0.01**
Amplitude	25.83 ± 0.73	25.81 ± 0.86	18.69 ± 0.74	19.20 ± 0.60	**<0.001**	**0.27**	0.547	<0.01	0.522	<0.01	**0.023**	**<0.01**
Density	2.50 ± 0.04	2.47 ± 0.04	2.22 ± 0.05	2.25 ± 0.06	**0.004**	**0.11**	0.882	<0.01	0.438	<0.01	**0.016**	**<0.01**
Duration	0.90 ± 0.01	0.90 ± 0.01	0.86 ± 0.01	0.87 ± 0.01	0.075 ^a^	0.04	0.853	<0.01	0.242	<0.01	0.056 ^a^	<0.01

Notes: Slow-wave and spindles detection was conducted with the default setting from SleepTrip toolbox on MATLAB R2024b. Prior to sleep spindles detection, individual slow and fast spindle frequency peaks were visually determined based on the NREM power spectrum of each dataset. Slow spindles peaks were determined in frontal channels (F3, F4) and fast spindles peaks in parietal channels (P3, P4) due to expected power maxima over those regions [11]. The algorithms used in SleepTrip are explained in detail in [7,8]. Values are means and standard errors of the mean. Significant results are highlighted in bold. ^a^ indicates *p* < 0.075. *η*^2^ represents the effect sizes where a value below or equal to 0.02 reflects a small effect, a value between 0.02 and 0.14 reflects a medium effect, and a value above 0.14 reflects a large effect.

## Data Availability

The original data presented in the study are openly available online in Open Science Framework at https://osf.io/a4vnb (accessed on 6 October 2025).

## Supplementary Materials

The following supporting information can be downloaded at: https://www.mdpi.com/article/10.3390/clockssleep7040068/s1.
