# Supplementary material for: Anticipation of Stress and Relaxation Dynamically Impacts Sleep
Source: Clocks Sleep. 2025 Dec 3;7(4):68. doi: 10.3390/clockssleep7040068 (PMC12731430; doi:10.3390/clockssleep7040068)
Supplement: Supplementary file 1 [file clockssleep-07-00068-s001.zip › clockssleep-3947009-supplementary.pdf]

Supplementary Material

for

Anticipation of stress and relaxation dynamically impacts sleep

Sandrine Baselgia[1], Jonas Beck[2] & Björn Rasch[1 *]

[1] Department of Psychology, Université de Fribourg, Fribourg, Switzerland
[2] Swiss Sleep House Bern, Department of Neurology, University Hospital (Inselspital), University of Bern, 3010 Bern, Switzerland

* Corresponding author:
Björn Rasch, University of Fribourg, Department of Psychology, Rue P.-A.-de-Faucigny 2, 1700 Fribourg, Switzerland

Tel. +41 26 300 7637, e-mail: bjoern.rasch@unifr.ch

Table S1: Post-hoc pairwise comparisons between conditions (stress vs relaxation/control) within studies for subjective and objective sleep parameters.

	Study I anticipated stress vs relaxation					Study II anticipated stress vs neutral control				
	**Stress**	**Control**	***t***	***p***	***d***	**Stress**	**Control**	***t***	***p***	***d***
**Subjective Sleep Parameters**										
Cognitive PSA	17.82 ± 1.03	16.18 ± 0.91	1.95	.060[a]	0.34	13.32 ± 0.72	13.52 ± 0.83	-0.25	.806	0.04
Somatic PSA	12.33 ± 0.70	12.27 ± 0.80	0.13	.898	0.02	16.74 ± 0.90	16.03 ± 0.94	0.77	.447	0.14
Sleep Quality	3.56 ± 0.14	3.57 ± 0.13	-0.05	.957	0.01	3.71 ± 0.12	3.65 ± 0.11	0.43	.670	0.08
SOL	23.18 ± 1.93	20.45 ± 1.97	1.33	.194	0.23	25.16 ± 1.97	22.58 ± 1.70	1.08	.290	0.19
**Objective Sleep Parameters**										
SOL [min]	16.76 ± 2.57	12.94 ± 1.74	**2.06**	**.047**	**0.36**	15.76 ± 1.67	19.15 ± 1.88	-1.87	.072[a]	0.34
WASO [%]	9.93 ± 2.63	5.74 ± 1.93	1.37	.179	0.24	8.10 ± 1.54	6.79 ± 1.86	0.55	.586	0.10
N1 [%]	20.05 ± 2.75	17.07 ± 2.22	1.53	.137	0.27	9.63 ± 1.13	11.30 ± 1.49	-1.14	.262	0.21
N2 [%]	37.82 ± 2.61	39.70 ± 2.57	-0.73	.470	0.13	44.92 ± 3.24	50.96 ± 3.55	-1.79	.083[a]	0.32
N3 [%]	20.55 ± 3.14	26.23 ± 3.20	-1.76	.088[a]	0.31	18.40 ± 3.37	12.21 ± 2.28	1.62	.116	0.29
REM [%]	11.64 ± 1.71	11.27 ± 1.77	0.18	.859	0.03	12.55 ± 2.17	12.37 ± 2.54	0.06	.954	0.01
TST [min]	63.67 ± 3.48	68.30 ± 3.16	-1.47	.150	0.26	64.81 ± 3.00	61.68 ± 2.96	0.86	.396	0.15
WASO [min]	7.18 ± 1.89	3.36 ± 0.85	1.94	.061[a]	0.34	5.84 ± 1.05	4.69 ± 1.20	0.73	.474	0.13
N1 [min]	13.14 ± 1.64	11.26 ± 1.35	1.27	.214	0.22	7.35 ± 0.80	7.55 ± 0.82	-0.23	.819	0.04
N2 [min]	26.50 ± 2.21	28.50 ± 2.07	-0.93	.357	0.16	33.95 ± 2.37	35.21 ± 1.52	-0.46	.651	0.08
N3 [min]	14.97 ± 2.52	19.80 ± 2.56	**-2.11**	**.043**	**0.37**	13.79 ± 2.23	9.40 ± 1.71	1.54	.135	0.28
REM [min]	9.06 ± 1.38	8.74 ± 1.42	0.19	.850	0.03	9.71 ± 1.70	9.45 ± 1.92	0.11	.916	0.02

Note: Subjective parameters are based on subjective ratings in the PSAS, assessing pre-sleep arousal (PSA) and SF-A/R questionnaire, measuring subjective sleep quality and sleep onset latency (SOL). Objective values are based on polysomnographic recordings. Sleep stages N1, N2, slow-wave sleep (SWS), rapid-eye movement (REM) sleep, wake after sleep onset (WASO), SOL, SWS latency, REM sleep latency are measured in minutes and percentages indicate partial percentage of total sleep time (TST). Values are mean and standard error of the mean. Significant results are highlighted in bold. [a] indicates $p < .09$. *d* represents the effect sizes where a value around 0.2 reflects a small effect, a value around 0.5 reflects a medium effect, and a value around 0.8 reflects a large effect.
